# Signaling through IL-17C/IL-17RE Is Dispensable for Immunity to Systemic, Oral and Cutaneous Candidiasis

**DOI:** 10.1371/journal.pone.0122807

**Published:** 2015-04-07

**Authors:** Heather R. Conti, Natasha Whibley, Bianca M. Coleman, Abhishek V. Garg, Jillian R. Jaycox, Sarah L. Gaffen

**Affiliations:** 1 University of Pittsburgh, Department of Medicine, Division of Rheumatology & Clinical Immunology, Pittsburgh, PA, United States of America; 2 Carnegie Mellon University, Dept. of Biological Sciences, Pittsburgh, PA, United States of America; King's College London Dental Institute, UNITED KINGDOM

## Abstract

*Candida albicans* is a commensal fungal microbe of the human orogastrointestinal tract and skin. *C*. *albicans* causes multiple forms of disease in immunocompromised patients, including oral, vaginal, dermal and disseminated candidiasis. The cytokine IL-17 (IL-17A) and its receptor subunits, IL-17RA and IL-17RC, are required for protection to most forms of candidiasis. The importance of the IL-17R pathway has been observed not only in knockout mouse models, but also in humans with rare genetic mutations that impact generation of Th17 cells or the IL-17 signaling pathway, including Hyper-IgE Syndrome (*STAT3* or *TYK2* mutations) or *IL17RA* or *ACT1* gene deficiency. The IL-17 family of cytokines is a distinct subclass of cytokines with unique structural and signaling properties. IL-17A is the best-characterized member of the IL-17 family to date, but far less is known about other IL-17-related cytokines. In this study, we sought to determine the role of a related IL-17 cytokine, IL-17C, in protection against oral, dermal and disseminated forms of *C*. *albicans* infection. IL-17C signals through a heterodimeric receptor composed of the IL-17RA and IL-17RE subunits. We observed that IL-17C mRNA was induced following oral *C*. *albicans* infection. However, mice lacking IL-17C or IL-17RE cleared *C*. *albicans* infections in the oral mucosa, skin and bloodstream at rates similar to WT littermate controls. Moreover, these mice demonstrated similar gene transcription profiles and recovery kinetics as WT animals. These findings indicate that IL-17C and IL-17RE are dispensable for immunity to the forms of candidiasis evaluated, and illustrate a surprisingly limited specificity of the IL-17 family of cytokines with respect to systemic, oral and cutaneous *Candida* infections.

## Introduction

The commensal fungus *Candida albicans* causes a wide spectrum of human pathologies, ranging from mild or chronic mucocutaneous infections to severe, often fatal disseminated disease. *Candida* colonization at mucosal surfaces or skin can progress to candidiasis in settings of immunosuppression, barrier disruption or broad-spectrum antibiotic use [1, 2]. Disseminated disease caused by *C*. *albicans* is the fourth most common nosocomial infection and is associated with a 20–80% mortality rate. HIV^+^/AIDS patients are especially vulnerable to oropharyngeal candidiasis (OPC, thrush), indicating the particular importance of CD4^+^ T cells in protection to this form of candidiasis [3]. Chronic mucocutaneous candidiasis (CMC) is a recurrent infection of skin, mucosae and nails (onychomycosis), frequently seen in individuals with mutations that affect Th17 cells, the cytokines IL-17A and IL-17F, or their receptor IL-17RA. Diseases associated with CMC include hyper-IgE syndrome (HIES, also known as Job’s syndrome) caused by mutations in *STAT3* or *TYK2*, or Autoimmune polyendocrinopathy syndrome (APS-1), caused by mutations in *AIRE*, or in genes impacting the IL-23/IL-17 signaling axis such as *IL12B*, *STAT1*, *IL17RA*, *IL17F*, and *ACT1* (reviewed in [4]).

In keeping with findings in humans, several studies demonstrated the importance of the IL-23/IL-17 pathway in protection to candidiasis in mice. We showed that IL-23-, IL-17RA-, IL-17RC- and Act1-deficient mice are all highly susceptible to OPC [5–8]. Similarly important roles for the IL-23/IL-17 signaling axis in controlling dermal and disseminated candidiasis in mouse models have also been described [9–11]. Collectively, these studies indicate that the mouse is a faithful model for understanding the nature of the immune response to *Candida* infections.

Biologic therapies targeting cytokines have shown considerable clinical efficacy in treating rheumatoid arthritis, psoriasis, Crohn’s disease and other autoimmune conditions. Currently there are FDA-approved monoclonal antibodies or soluble receptors that directly or indirectly block the Th17/IL-17 axis, including agents that neutralize TNF-α, IL-1β, IL-6R and IL-12/23p40. More recently, Phase II and III clinical trials testing biologics that directly target IL-17A or IL-17RA have shown considerable promise in treating psoriasis, and are under evaluation for a number of other rheumatologic conditions [12–16]. Given the accumulating genetic evidence that the IL-17RA/RC complex is essential for protection from candidiasis, it is important to understand the potential adverse side effects of anti-IL-17/IL-17RA therapy on immune responses that protect against opportunistic infections.

The IL-17 cytokine family consists of six related members: IL-17A, IL-17B, IL-17C, IL-17D, IL-17E (IL-25) and IL-17F [17, 18]. To date, almost nothing is known about the antifungal functions of IL-17 family cytokines apart from IL-17A and IL-17F. IL-17A and IL-17F signal through a heterodimeric receptor composed of IL-17RA and IL-17RC [19, 20]. In addition, IL-17RA partners with other members of the extended IL-17 receptor family to form binding complexes for other IL-17-family cytokines [18], and thus is considered the common signaling subunit of the IL-17 family. In particular, the cytokine IL-17C signals through a receptor complex containing IL-17RA paired with IL-17RE [21]. In contrast to IL-17A and IL-17F, which are lymphocyte-derived, IL-17C is predominantly produced by epithelial cells and keratinocytes [21, 22]. Similar to IL-17A, IL-17C directs an immune response at mucosal surfaces and skin by stimulating production of pro-inflammatory cytokines, chemokines and antimicrobial peptides. The downstream genes induced by IL-17C show overlap with those regulated by IL-17A [23] [24, 25]. It has been suggested that IL-17C amplifies the Th17 response by direct signaling on Th17 cells through IL-17RE/IL-17RA [26]. Several studies have indicated a protective role for IL-17C in gut and skin [27–29], but this cytokine is still remarkably poorly understood. Because so little is known about the antifungal roles of other non-IL-17A family members such as IL-17C, and because IL-17C signals through the shared receptor IL-17RA, we sought to determine the role of the IL-17C/IL-17RE signaling axis in immunity using three standard models of infectious candidiasis. Surprisingly, however, we detected no role for the IL-17C/IL-17RE signaling axis in these forms of experimental candidiasis.

## Materials and Methods

### Mice

C57BL/6 mice were from The Jackson Laboratory (Bar Harbor, Maine). IL-17RA^-/-^ mice were a kind gift from Amgen (Seattle, WA). IL-17RC^-/-^, IL-17C^-/-^ and IL-17RE^-/-^ mice were kindly provided by Genentech (South San Francisco), produced in collaboration between Genentech and Lexicon Pharmaceuticals to analyze the function of 500 secreted and transmembrane proteins [30]. All mice were bred at the University of Pittsburgh under a 12 hour light/dark cycle. IL-17C^-/-^ and IL-17RE^-/-^ mice are on a mixed genetic background, so littermate controls were used for experiments, as noted. Genotypes were verified for all animals by PCR of ear biopsies. WT cohorts consisted of in-house bred littermate controls or C57BL/6 mice from The Jackson Laboratory as appropriate for each experiment, with sample sizes based on power analyses calculated from previously published data [31]. Cohorts were selected randomly and were age- and sex-matched using both males and females at a range of 6–10 weeks. Mice were housed in SPF conditions and provided with autoclaved food and water *ad libitum*. Mice were monitored visually and weighed at least once daily. Mice were humanely sacrificed by CO_2_ inhalation at the termination of each experiment or if animals exhibited >20% weight loss or showed signs of pain or distress as delineated by the approved animal protocol. The University of Pittsburgh Institutional Animal Care and Use Committee (IACUC) approved all animal protocols used in this study (Animal welfare assurance number: A3187-01). All efforts were made to minimize suffering, in accordance with recommendations in the Guide for the Care and Use of Laboratory Animals of the National Institutes of Health.

### Oropharyngeal Candidiasis

Mice were pre-swabbed orally prior to each experiment to verify the absence of pre-existing *Candida* colonization. Mice were inoculated sublingually for 75 mins under anesthesia (ketamine 100 mg/kg and xylazine 10 mg/kg) *Candida albicans* (strain CAF2-1) placed in an sterile saturated 0.0025 mg cotton ball, as previously described [6, 32]. WT littermates were used as a negative control as they are known to clear the infection fully by day 5 post-inoculation [6, 31]. At the end of the designated time course (4–5 d), tongue was homogenized using a Miltenyi GentleMacs Dissociator (Miltenyi Biotec). Serial dilutions were plated in triplicate on YPD agar plates, incubated at 30°C for 48 h, and colony-forming units (CFU) were enumerated for tissue fungal burden determination. Mice were weighed daily. Mice were sacrificed for humane reasons if they lost more than 25% weight loss or exhibited other signs of pain or distress (however, in these experiments, no animals fell in this category). Each dot represents one mouse. There were no severe adverse events in any group.

### Disseminated Candidiasis

*C*. *albicans* (strain SC5314) was grown overnight in YPD at 30°C with continuous agitation. Age- and sex-matched mice were injected in the tail vein with 1-2x10^5^ *C*. *albicans* cells suspended in 100ul PBS, as described [31, 33]. For injections, mice were briefly held in a commercial restraining apparatus (Braintree Scientific, Braintree MA). After 10 d or when when weight loss exceeded 20% or showed other signs of distress such as severe hunching, shivering or loss of righting, mice were humanely sacrificed by C0_2_ inhalation followed by cervical dislocation. There were no unexpected adverse events in any group. Kidney tissue was harvested and homogenized using C-tubes (Millipore) in 1 ml PBS. Homogenates were diluted and plated on YPD-AMP agar in triplicate and CFU enumerated for fungal burden determination.

### Cutaneous Candidiasis

A suspension of *C*. *albicans* (strain CAF2-1) was cultured overnight in YPD agar with shaking at 30°C. Two hours before infection the *C*. *albicans* suspension was diluted to 5x10^6^ cells/ml and cells were transferred to YPD broth containing 10% FBS and incubated with shaking to induce hyphal formation. A 95% conversion to hyphae was confirmed microscopically. Mice were intradermally inoculated with 50 ul of *Candida* hyphae in PBS as described previously (ref). Mice were scored by blinded evaluators at least 3 times per week for the presence or complete absence of 4 clinical parameters: ulceration, crusting, erythema and nodule formation. Mice were considered negative for healing if at least one parameter was present. There were no severe adverse events in any group.

### Real-time PCR

Total RNA was isolated using RNeasy Mini Kits (Qiagen). cDNA synthesis was performed using Superscript III First Strand kits (Invitrogen, Carlsbad CA). Genes were measured by real time-reverse transcriptase PCR (qPCR) using SYBR Green FastMix ROX from Quanta Biosciences (Gaithersburg, MD). PCR reactions were performed on a 7300 Real Time PCR Systems instrument (Applied Biosystems, CA). Expression was normalized to *Gapdh*. Primers were from Super Array Biosciences or QuantiTect Primer Assays (Qiagen).

### Statistics

Data were analyzed by Kaplan-Meier, ANOVA, Mann-Whitney or unpaired Student's *t* test using GraphPad Prism (La Jolla, CA). *P* values <0.05 were considered significant. All experiments were performed a minimum of twice to ensure reproducibility.

## Results

### IL-17C/ and IL-17RE are not required for protection against oropharyngeal candidiasis (OPC)

**Fig 1:**
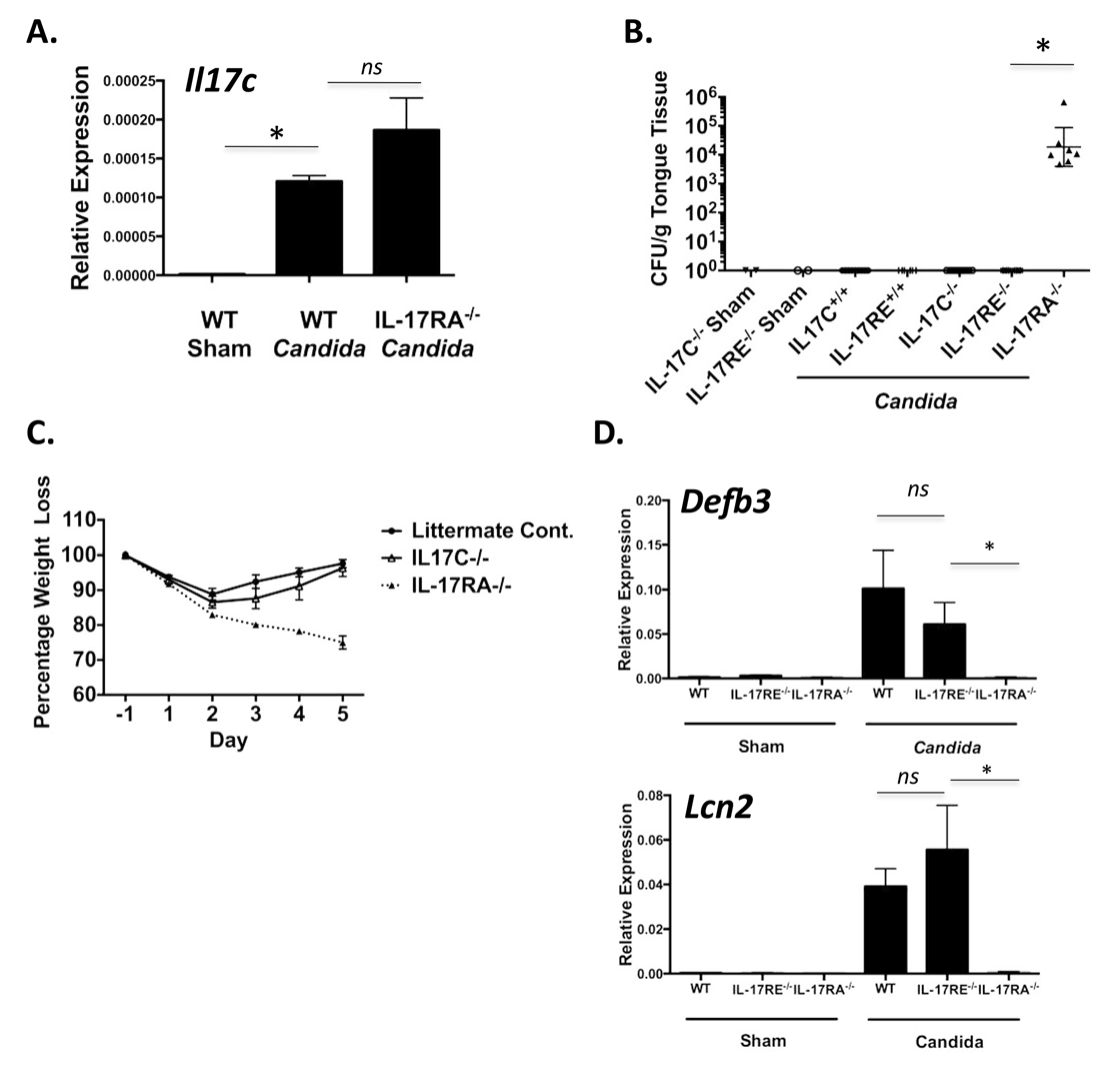
IL-17C^-/-^ and IL-17RE^-/-^ mice are resistant to oropharyngeal candidiasis. **A.** WT and IL-17RA^-/-^ mice (n = 3 per group) were subjected to OPC and IL-17C transcript levels assessed by qPCR. *p<0.05 with error bars indicating SEM. **B.** OPC was induced in the indicated mice (IL-17C^-/-^ Sham, n = 2; IL-17RE^-/-^ Sham, n = 2; Infected: IL-17C^+/+^ n = 9; IL-17RE^+/+^, n = 6; IL-17C^-/-^, n = 9; IL-17RE^-/-^, n = 6; IL-17RA^-/-^ n = 7). Tongue was harvested on day 5, and CFU enumerated 48 h later by plating serial dilutions on YPD agar. Data presented as the geometric mean of CFU. Each data point represents one mouse, and the graph depicts pooled data from two independent experiments (n = 2 for each sham-infected cohort and n≥6 total for each Candida-infected cohort). *p<0.0001 by Mann Whitney U test. **C.** Weight loss was assessed daily and is presented as a percentage of starting weight. Error bars indicate SEM. **D.** Indicated mice (n = 3 per group) were subjected to OPC and qPCR performed for Defb3 and Lcn2 genes on day 2. *p<0.05 by ANOVA and post-hoc Tukey’s test. Error bars indicate SEM.

As a first step to determining whether IL-17C participates in immunity to OPC, we determined whether this cytokine was induced following oral *C*. *albicans* infection. WT mice were inoculated orally with *C*. *albicans* (CAF2-1) for 75 min under anesthesia per a standard protocol used widely in the literature [32, 34]. As shown (Fig 1A), mRNA encoding IL-17C was not detectable at baseline but was markedly induced 2 days post infection. *Il17c* mRNA was also elevated in IL-17RA^-/-^ mice subjected to OPC, indicating that its expression is not downstream of IL-17R signaling. This finding is consistent with published results showing that *Il17a* mRNA is also strongly induced 2 days post-OPC [5, 35].

The fact that a gene is induced during an infection does not necessarily mean that it is required for effective immunity to that organism. Therefore, to determine whether IL-17C or its receptor IL-17RE were needed to mediate protection against OPC, IL-17C^-/-^ and IL-17RE^-/-^ mice were subjected to OPC and the oral fungal load was assessed after 5 days. Like healthy humans, WT mice are able to clear the infection fully within 5 days of exposure to *C*. *albicans*. Although immunosuppression with corticosteroids is often used as a positive control in this model of OPC, we instead employed IL-17RA^-/-^ mice as controls (without additional immunosuppression), since these mice are reproducibly susceptible to disease, judged by both fungal burden and progressive weight loss [6, 8]. Indeed, as we have seen in numerous prior studies, 5 days post infection IL-17RA^-/-^ mice exhibited a high fungal burden (~1x10^4^ CFU/g tissue) and showed a 20% weight loss, validating the importance of IL-17RA signaling in protection against OPC [6, 8] (Fig 1B and 1C). In contrast, IL-17C^-/-^ and IL-17RE^-/-^ mice completely cleared the infection by day 5, with no detectable fungal burden in the tongue (Fig 1B). Moreover, IL-17C^-/-^ and IL-17RE^-/-^ mice fully regained weight after a transient weight loss due to the infection procedure, in a pattern identical to WT mice (Fig 1C and data not shown). Therefore, whereas IL-17RA^-/-^ mice were highly susceptible to OPC, IL-17C- and IL-17RE-deficient mice were fully resistant.

A strong neutrophil response and production of antimicrobial proteins protect immunocompetent mice from *C*. *albicans* infection [1, 6, 36, 37]. We previously identified a panel of IL-17RA-dependent signature genes that are induced after exposure to *C*. *albicans* that represent the overall transcriptional response to infection [6]. Most of these genes are regulated by IL-17RA signaling, including *Lcn2* (encoding lipocalin-2, also known as 24p3) and *Defb3* (encoding β-defensin 3, also known as BD3), an antimicrobial peptide with direct anti-*Candida* activity [38, 39]. Consistent with their resistance to infection, WT littermate controls, IL-17C^-/-^ and IL-17RE^-/-^ mice all exhibited similar gene profiles after inoculation, and representative genes such as *Defb3* and *Lcn2* were strongly induced in each cohort (Fig 1D). In contrast, these transcripts were detected at significantly lower levels in IL-17RA^-/-^ mice (Fig 1D), consistent with our prior findings [6, 35]. In conclusion, these data indicate that, while IL-17A/IL-17RA signaling is essential for protection to OPC, IL-17C/IL-17RE signaling appears to be dispensable.

### IL-17C- and IL-17RE-deficient mice are resistant to disseminated candidiasis

**Fig 2:**
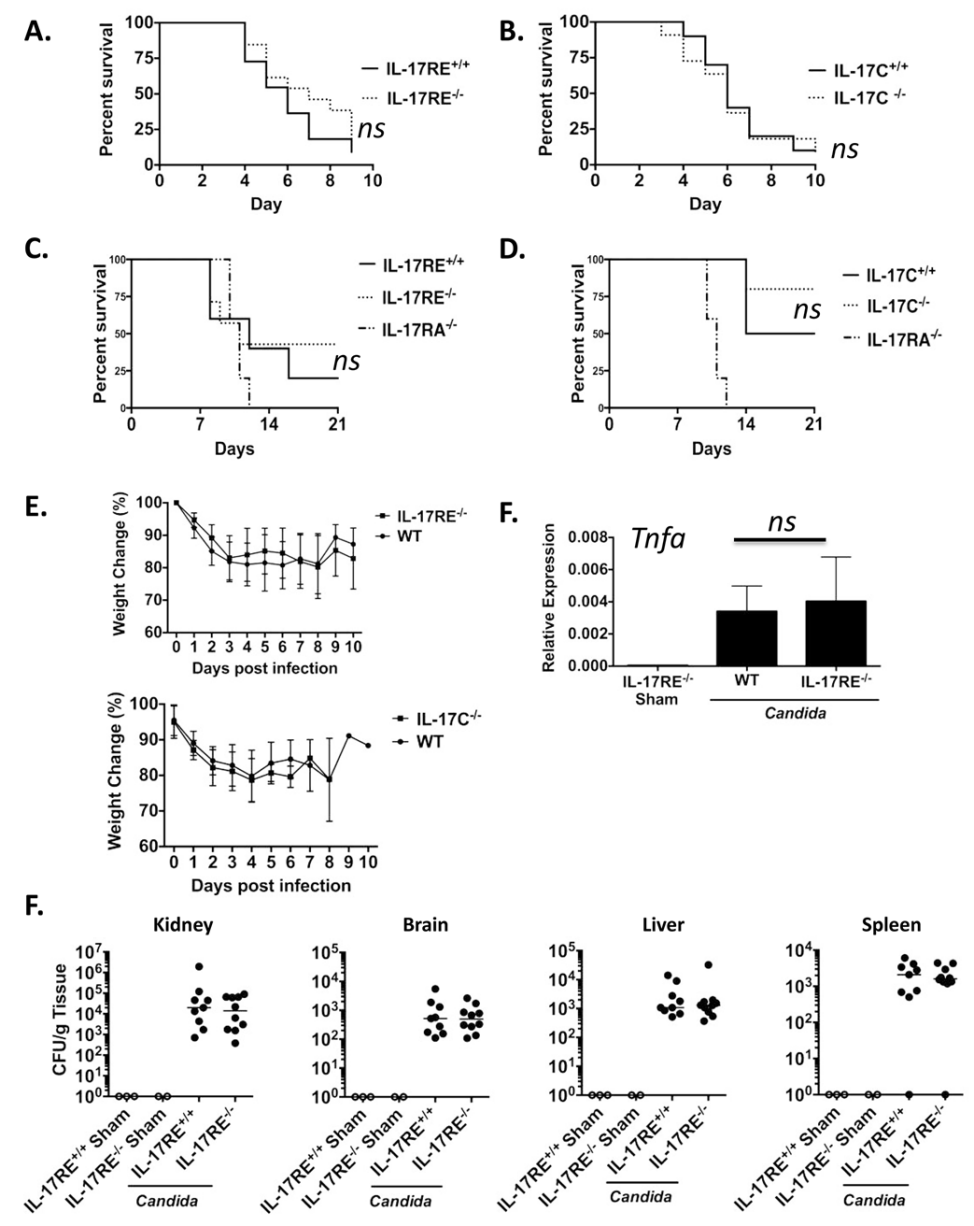
IL-17C^-/-^ and IL-17RE^-/-^ mice are resistant to systemic candidiasis. **A-C.** IL-17RE^-/-^ and IL-17RE^+/+^ littermate controls were subjected to systemic candidiasis by tail vein injection of 2x10^5^ C. albicans strain SC5314 (IL-17RE^+/+^ n = 11; IL-17RE^-/-^, n = 13; IL-17C^+/+^; n = 10; IL-17C^-/-^ n = 11). Mice were evaluated daily and time to sacrifice is presented. ns., not significant. Data are representative of 2 independent experiments. **C-D.** IL-17RE^-/-^ mice (n = 7) and IL-17C^-/-^ (n = 5) mice were injected with 1x10^5^ cells C. albicans as in panel A. WT littermates (IL-17C^+/+^, n = 4 and IL-17RE^+/+^, n = 5) and IL-17RA^-/-^ mice (n = 5) served as controls. ns., not significant. Data are representative of one independent experiment. **E.** Weight change assessments were made in all cohorts after infection with 2x10^5^ C. albicans cells, and presented as a percentage of starting weight. **F.** Kidneys were harvested from each cohort on day 2 after systeic infection and qperformed for the indicated genes. *p<0.05 by ANOVA and post-hoc Tukey’s test. **G.** Indicated tissue types were harvested on day 2 and tissue fungal burdens determined. Each data point represents one mouse. (IL-17RE^+/+^ Sham, n = 3; IL-17RE^-/-^ Sham, n = 2; IL-17RE^+/+^ n = 9; IL-17RE^-/-^ n = 10). Pooled data from two independent experiments is shown.

In addition to a critical role in protecting against oral mucosal candidiasis, IL-17RA and IL-17A are protective in an intravenous model of disseminated candidiasis [9, 10, 40]. We therefore assessed the importance of IL-17C and IL-17RE in protection from systemic candidiasis by infecting mice in the tail vein with a standard dose of 2x10^5^ *C*. *albicans* yeast cells (strain SC5314, the strain most commonly employed in this model and which induces the same level of disease as strain CAF2-1). As shown, there was no apparent role for IL-17C or IL-17RE signaling in host defense against systemic candidiasis, as IL-17C^-/-^ and IL-17RE^-/-^ mice followed the same survival curve as littermate control mice (Fig 2A and 2B). The mice showed identical weight loss profiles throughout the experiment (Fig 2C), and the mice did not exhibit any other symptoms of disease that differed from the WT littermate controls (HRC and NW, unpublished observations). We also infected mice with a lower inoculum of *C*. *albicans* (1x10^5^ yeast cells), in order to rule out a protective effect for IL-17C and IL-17RE in less severe disease. Again, IL-17C^-/-^ and IL-17RE^-/-^ mice demonstrated the same susceptibility to disseminated disease as WT littermate controls, while IL-17RA^-/-^ were reproducibly more susceptible to infection (Fig 2C and 2D) [10]. IL-17RE-deficient and WT mice also exhibited similar induction of TNFα after infection, a cytokine known to be important in mediating immunity to systemic candidiasis (Fig 2E). Dissemination of *C*. *albicans* to visceral organs including brain, kidney, liver and spleen was also similar in control mice and IL-17C^-/-^ and IL-17RE^-/-^ mice (Fig 2F). These data indicate that, in contrast to IL-17A and IL-17RA, IL-17C and IL-17RE are not required for protection in disseminated candidiasis.

### IL-17C and IL-17RE signaling are not required for resolution of cutaneous candidiasis

**Fig 3:**
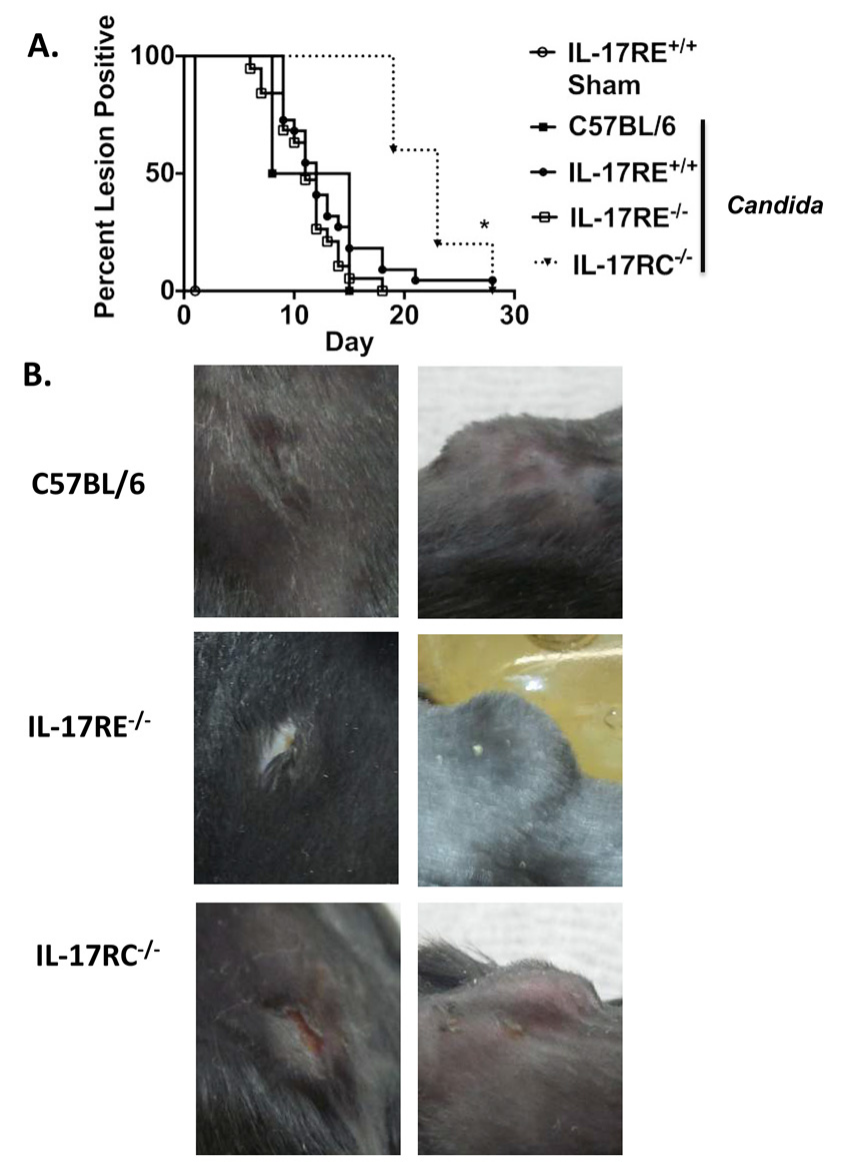
IL-17RE^-/-^ mice are resistant to cutaneous candidiasis. **A.** The indicated mice were subjected to dermal candidiasis by subcutaneous injection with C. albicans strain CAF2-1 hyphae (IL-17RE^+/+^ Sham, n = 6; Infected: IL-17RC^-/-^, n = 5; C57BL/6 WT, n = 4; IL-17RE^+/+^, n = 21; IL-17RE^-/-^, n = 19). The percent of mice positive for lesions over time is presented. *p<0.01 compared to WT by a Log-rank (Mantel Cox) test. Graph depicts pooled data from two independent experiments. **B.** Ulceration (left panels) and nodule formation (right panels) are depicted in WT, IL-17RE^-/-^ and IL-17RC^-/-^ mice on days 4 and 6. Data are representative of two independent experiments.

Since IL-17C and IL-17RE are expressed in the skin and have been shown to be pathogenic in mouse models of dermal inflammation [23, 29], we hypothesized that the most likely setting in which the IL-17C/IL-17RE axis might participate in antifungal immunity would be in cutaneous *C*. *albicans* infections. Accordingly, IL-17RE^-/-^ and littermate control mice were infected intradermally with *C*. *albicans* hyphae (strain CAF2-1) using an infection model that previously demonstrated an essential of IL-17RA in protection from dermal candidiasis [11]. In this model, the most consistent readout for disease progression is the rate of healing at the site of injection. In the OPC model fungal tissue burden is highly consistent between replicates and hence a good indicator of disease progression [32, 34]; in contrast, in the cutaneous model, fungal burden determination and histology are highly variable and therefore are not considered a reliable measure of disease. Accordingly, we scored mice for healing at the site of injection based on four clinical parameters: (i) nodule formation, (ii) ulceration, (iii) crusting and (iii) erythema, as previously described [11, 31]. A mouse scored negatively for healing if at least one symptom was noted. The IL-17RE^-/-^ and WT littermate control mice followed indistinguishable healing curves, indicating that they are resistant to cutaneous candidiasis. Since it was shown previously that IL-17RA is necessary for protection to dermal candidiasis, we tested susceptibility in IL-17RC^-/-^ mice, lacking the essential co-receptor for IL-17A signaling [8, 19, 41]. In contrast to IL-17RE^-/-^ mice, IL-17RC^-/-^ mice showed significantly delayed healing (Fig 3A and 3B), consistent with the importance of IL-17RA and IL-17A for immunity to dermal disease [11]. Fungal loads were also elevated in IL-17RC^-/-^ mice compared to WT mice (data not shown). The similarities between WT littermate controls and IL-17RE^-/-^ mice indicate that IL-17C signaling via IL-17RE is not essential for protection from or the resolution of cutaneous candidiasis, whereas IL-17A signaling through IL-17RA/IL-17RC is nonredundant.

## Discussion

IL-17A came into prominence with the discovery of the Th17 subpopulation of T cells [42]. Subsequent work from our group and others showed that the IL-17RA subunit is essential for immunity to various forms of candidiasis, not only in experimental mouse models but also in humans with genetic predispositions to CMC [6, 10, 11, 43]. A similar role in anti-*Candida* immunity was found for IL-17RC, the obligate co-receptor for IL-17A-mediated signaling [8, 19]. Comparisons of IL-17A and IL-17F generally indicate that IL-17A is more potent than IL-17F in mediating immunity to candidiasis, though this issue is still not fully defined [40, 44]. Although the importance of IL-17RA/RC was clear, IL-17RA is used by other ligands including IL-17C, leaving open the possibility that cytokines in addition to IL-17A might contribute to immunity to candidiasis.

Of the other IL-17 family members, we considered that IL-17C and its receptor IL-17RE were the most likely to be important in candidiasis, given the similarity of the IL-17A- and IL-17C-induced downstream gene profiles and their common propensity to act at mucosal surfaces. Indeed, *Il17c* mRNA was strongly induced following *Candida* infection (Fig 1), in a manner similar to *Il17a* mRNA [5]. Therefore, it was surprising that IL-17C/IL-17RE signaling was dispensable for the three forms of candidiasis evaluated here. Perhaps the most unexpected result was the absence of a role for IL-17C or IL-17RE in dermal candidiasis, since IL-17C is pathogenic when overexpressed transgenically in skin [29], and elevated IL-17C is observed in human psoriatic lesions [29, 45]. It may be that the effects of IL-17C are manifested predominantly in autoimmune disease rather than during infection settings. Along these lines, IL-17C^-/-^ mice are partially protected from experimental autoimmune encephalomyelitis (EAE), the mouse model of multiple sclerosis and DSS colitis [22, 26, 46]. IL-17C is also associated with murine arthritis models [24]. In terms of host defense, IL-17C has been implicated in protection against *C*. *rodentium* infections in the rodent GI tract, although its role in regulating intestinal colonization of fungal species is unknown [21].

In this study, we used the most widely accepted models of oral, dermal and cutaneous candidiasis, none of which revealed a role for IL-17C or IL-17RE in host defense. However, other variations of these models exist that employ other strains of *Candida albicans*, genetic backgrounds, or slightly different routes of *Candida* delivery, where it is possible that a role for IL-17C/IL-17RE might be observed. Similarly, there may be a role for IL-17C/IL-17RE signaling in other types of fungal infections. The role of IL-17 in vaginal candidiasis is controversial, but some evidence suggests Th17 cells may contribute to immunity at this site [47, 48]. Clearly, future studies to define the biological activities of IL-17C signaling in vaginal candidiasis are warranted.

IL-17A and IL-17F are produced by multiple cell types. Conventional Th17 cells are the most well known source, but CD8^+^ cells also express IL-17. More recently, important innate sources of IL-17A have been identified, including γδ-T cells, ‘natural’ Th17 (nTh17) cells and group 3 innate lymphoid cells (ILC3) [49, 50]. In humans, the dominant CD4^+^ T cell response to *C*. *albicans* occurs in the Th17 compartment [51], although the role of innate IL-17^+^ cells in humans are not well understood. Similarly, in a mouse model of OPC, CD4^+^ Th17 cells are generated in mice following a re-challenge infection [35, 52, 53]. In naïve settings, the dominant source of IL-17A during a primary oral *Candida* infection in mice comes from γδ-T cells and nTh17 cells [5]. In skin, IL-17A also comes from γδ-T cells in mouse models [11, 54, 55]. To date, the source of IL-17A during systemic candidiasis is not well defined in mice or humans, but is thought to be innate. IL-17C, in contrast, is produced by epithelial cells, not by lymphocytes [25]. The induction of *Il17c* mRNA in the oral mucosa during OPC (Fig 1) was the impetus for this study, but despite its induction at the mRNA level, mice lacking IL-17C were resistant to the forms of candidiasis tested including OPC. However, this is not necessarily unexpected; a number of inflammatory factors are induced in OPC that are not required for immune protection. For example, lipocalin 2 is a potent IL-17-target gene that is highly upregulated in *Candida*-infected tongue tissue, but is not required for mediating immunity to OPC, as *Lcn2* ^-/-^ mice are resistant to infection [7, 56]. Thus, this work identifies another setting in which induction of a gene (*Il17c*) does not necessarily imply a non-redundant role in immunity.

IL-17A and IL-17RA are emerging as effective biologic targets for autoimmune conditions, particularly psoriasis [14]. On the flip side, the clinical blockade of IL-17A or IL-17RA signaling is likely to cause complications for IL-17A-directed antifungal immune responses. Data from individuals with mutations that affect the Th17/IL-17R pathway have provided evidence that IL-17 deficiencies lead to CMC in humans [4]. A direct requirement for IL-17 was demonstrated in a patient with a homozygous mutation in *IL17RA* who presented with autosomal recessive CMC [43]. Similarly, two individuals with mutations in *ACT1*, a signaling adaptor almost entirely restricted to the IL-17 family [17], also experienced CMC [57]. More relevant to clinical treatment, neutralizing autoantibodies against IL-17A and other Th17 cytokines (IL-17F, IL-22) are produced by APS-1 patients, resulting in selective susceptibility to CMC manifestations including OPC [58, 59]. It is not yet clear to what extent therapeutic manipulation of the IL-17RA pathway will cause candidiasis, but this issue is obviously of clinical concern [16]. It is striking, however, that no patients with CMC have yet emerged with defects in the IL-17C/IL-17RE pathway, whereas an ever-increasing number have been found that are impaired in the IL-17A/F-IL-17RA pathway [4]. Thus, the present study raises the possibility that future therapeutics for psoriasis could potentially target IL-17C selectively with a lesser risk of susceptibility to *C*. *albicans*.

## Supporting Information

S1 Checklist ARRIVE Checklist. (PDF)
